# Coordination Chemistry inside Polymeric Nanoreactors: Metal Migration and Cross-Exchange in Amphiphilic Core-Shell Polymer Latexes

**DOI:** 10.3390/polym8020026

**Published:** 2016-01-22

**Authors:** Si Chen, Eric Manoury, Florence Gayet, Rinaldo Poli

**Affiliations:** 1 CNRS, LCC (Laboratoire de Chimie de Coordination), Université de Toulouse, UPS, INPT, 205 Route de Narbonne, BP 44099, F-31077 Toulouse Cedex 4, France; si.chen.yang@hotmail.com (S.C.); eric.manoury@lcc-toulouse.fr (E.M.); florence.gayet@lcc-toulouse.fr (F.G.); 2 Institut Universitaire de France, 103, bd Saint-Michel, 75005 Paris, France

**Keywords:** core cross-linked micelles, interparticle molecular migration, phosphine-functionalized polymers, polymer latex, RAFT polymerization, rhodium complexes

## Abstract

A well-defined amphiphilic core-shell polymer functionalized with bis(*p*-methoxy-phenylphosphino)phenylphosphine (BMOPPP) in the nanogel (NG) core has been obtained by a convergent RAFT polymerization in emulsion. This BMOPPP@NG and the previously-reported TPP@NG (TPP = triphenylphosphine) and core cross-linked micelles (L@CCM; L = TPP, BMOPPP) having a slightly different architecture were loaded with [Rh(acac)(CO)_2_] or [RhCl(COD)]_2_ to yield [Rh(acac)(CO)(L@Pol)] or [RhCl(COD)(L@Pol)] (Pol = CCM, NG). The interparticle metal migration from [Rh(acac)(CO)(TPP@NG)] to TPP@NG is fast at natural pH and much slower at high pH, the rate not depending significantly on the polymer architecture (CCM *vs.* NG). The cross-exchange using [Rh(acac)(CO)(BMOPPP@Pol)] and [RhCl(COD)(TPP@Pol)] (Pol = CCM or NG) as reagents at natural pH is also rapid (*ca.* 1 h), although slower than the equivalent homogeneous reaction on the molecular species (<5 min). On the other hand, the subsequent rearrangement of [Rh(acac)(CO)(TPP@Pol)] and [RhCl(COD)(TPP@Pol)] within the TPP@Pol core and of [Rh(acac)(CO)(BMOPPP@Pol)] and [RhCl(COD)(BMOPPP@Pol)] within the BMOPPP@Pol core, leading respectively to [RhCl(CO)(TPP@Pol)_2_] and [RhCl(CO)(BMOPPP@Pol)_2_], is much more rapid (<30 min) than on the corresponding homogeneous process with the molecular species (>24 h).

## 1. Introduction

The use of hierarchically-organized polymers as catalyst supports (catalytic nanoreactors) is an emerging area [1,2]. Of particular interest in our group are unimolecular assemblies rather than self-assembled reversible micellar objects, because their swelling by solvents and reagents/products remains limited, keeping the nanoreactor size under better control and, thus, avoiding undesired phenomena, such as the formation of stable emulsions. In addition, the absence of free arm-micelle equilibrium reduces the degree of catalyst leaching. Unimolecular polymeric nanoreactors have been obtained by cross-linking linear polymers, after self-assembly in micellar form, at the level of either the outer shell [3,4,5,6], the inner core [7,8,9,10] or an intermediate corona [11,12]. Nanoreactors with a nanogel core have also been obtained by direct functionalization and cross-linking starting from linear polymers as macroinitiators [13,14,15,16,17,18,19,20]. Although a few of these nanoreactors have been used in combination with transition metal complexes as pre-catalysts for a variety of transformations, details of the pre-catalyst bonding to the polymer scaffold (coordination environment, stability, mobility) have typically not been looked at.

**Figure 1:**
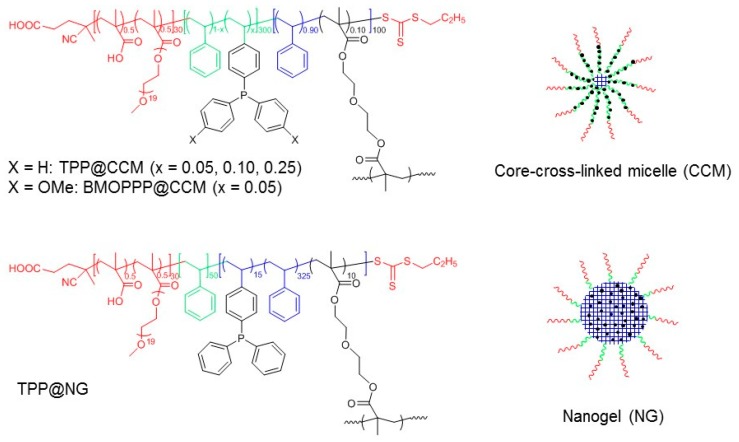
Catalytic nanoreactors previously made in our laboratory [7,8,9,10].

We have recently applied catalytic nanoreactors and, more specifically, phosphine ligand-functionalized core-cross-linked micelles (CCM) made in our laboratory, for the first time, to aqueous biphasic catalysis based on the micellar catalysis principle, namely with the catalytic act taking place within the hydrophobic core of the water-phase-confined polymer and with the catalytic phase being recovered by decantation [7,8,9]. This operating protocol differs from those previously applied in nanoreactor catalysis, which used either homogeneous conditions with catalyst recovery by precipitation/filtration or by ultrafiltration or aqueous biphasic conditions with separation/recovery by decantation, but with the catalytic act occurring either in the organic phase at high temperature by the thermomorphic approach [21,22] or at the water/organic interface [23,24,25,26,27]. The structure of the CCM polymers, made by a convergent one-pot three-step procedure using reversible addition-fragmentation chain transfer (RAFT) polymerization [28,29,30,31,32,33] through the “polymerization-induced self-assembly” (PISA) approach in aqueous dispersed media [34,35], is shown in Figure 1.

**Figure 2:**
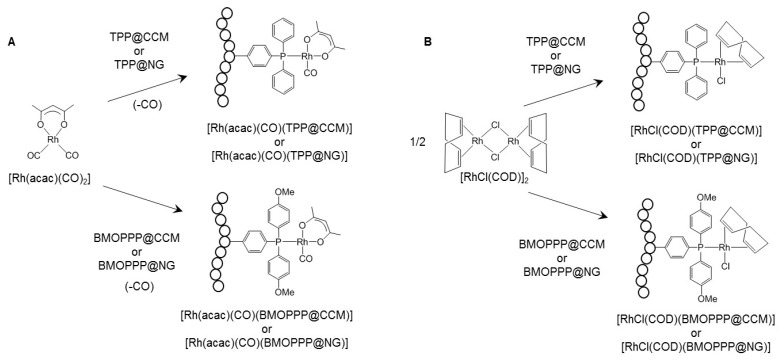
Coordination of the pre-catalyst to the polymeric nanoreactors.

The catalytic transformation scrutinized with these nanoreactors, after loading with the [Rh(acac)(CO)_2_] pre-catalyst (acac = acetylacetonato), was the hydroformylation of 1-octene as a representative example of a water-insoluble higher α-olefin. This is a catalytic transformation of strong industrial relevance with a >12 Mtons annual production worldwide [36,37,38,39,40,41]. The pre-catalyst is transformed to polymer-linked [Rh(acac)(CO)(L@CCM)] (L = triphenylphosphine, TPP [7,9] or bis(*p*-methoxyphenyl)phenylphosphine, BMOPPP [8]) prior to the catalytic reaction (Figure 2A) and then to a polymer-linked [RhH(CO)_4−*n*_(phosphine)*_n_*] active catalyst upon interaction with syngas (H_2_/CO mixture) during catalysis. We have more recently synthesized and applied to aqueous biphasic hydroformylation catalysis nanoreactors having a slightly different architecture, called nanogels (NG), functionalized with triphenylphosphine (TPP@NG); see Figure 1 [42]. These nanoreactors are characterized by a fully cross-linked core, and their overall composition is identical to those of TPP@CCM (x = 0.05) and BMOPPP@CCM. The cross-linker and the functionalized monomer were added simultaneously in Step 3, after a short chain extension with styrene in Step 2.

Spectroscopic (^31^P NMR) investigations of metal coordination, related to the fundamental question of metal mobility between different nanoreactors, have led to the discovery of a very rapid interparticle metal migration process that occurs through a direct associative phosphine exchange during reversible interpenetration with core-core contact [43]. These experiments were carried out by mixing together, after core swelling with toluene, equimolar amounts of metal-free and fully-metal-loaded TPP@CCM particles. Even though the metal does not change the coordination environment when moving from one nanoreactor to another, these experiments gave unambiguous information on the rate of metal migration because of the rapid self-exchange process taking place within 50% loaded particles; see Equation (1). Hence, while the fully-loaded particles show a ^31^P resonance as a doublet at δ 47.5 (*J*_PRh_ = 175 Hz) and the metal-free particles show a single resonance at δ −6.6, the fully-exchanged (50% loaded) final sample has a silent ^31^P NMR spectrum, because the rate of the interparticle degenerative exchange of Equation (1) gives signal coalescence at room temperature [43]. Mixing at the natural pH (*ca.* 5) resulted in immediate signal disappearance, whereas a much slower (>10 h) metal migration occurred at pH 13.6 because of the particle Coulombic repulsion by the deprotonated shell methacrylic acid functions. Preliminary studies, to be fully described elsewhere, indicate that the pre-catalyst stability and the catalytic activity are not affected by pH.

TPP@CCM + [Rh(acac)(CO)(TPP@CCM)] ⇄ [Rh(acac)(CO)(TPP@CCM)] + TPP@CCM
(1)

The current report expands on the investigation of the metal migration process by addressing the following variations: (i) migration of the [Rh(acac)(CO)] fragment for the nanogel (TPP@NG) particles and comparison with TPP@CCM to assess the role of the polymer architecture on the particle interpenetration process; (ii) metal cross-exchange between differently-functionalized polymers, using both the CCM and the NG particles, to remove the rapid intraparticle ligand exchange of Equation (1). The cross-exchange uses TPP and BMOPPP as ligands and [Rh(acac)(CO)] and [RhCl(COD)] (COD = η^4^-1,5-cyclooctadiene) as metal fragments. The latter investigation required the synthesis of BMOPPP@NG and the independent study of the coordination chemistry of the [RhCl(COD)] fragment (obtained by bridge-splitting from the corresponding dimer; Figure 2B), which had not been previously reported.

## 2. Materials and Methods

### 2.1. General

All manipulations were performed under an inert atmosphere of dry argon by using Schlenk line techniques. Solvents were dried by standard procedures and distilled under argon prior to use. 4,4′-azobis(4-cyanopentanoic acid) (ACPA, >98%, Fluka, Saint-Quentin Fallavier, France), methacrylic acid (MAA, 99.5%, Acros, Illkirch, France), poly(ethylene oxide) methyl ether methacrylate (PEOMA, *M*_n_ = 950 g·mol^−1^, Aldrich, Saint-Quentin Fallavier, France), di(ethylene glycol) dimethacrylate (DEGDMA, 95%, Aldrich), 1,3,5-trioxane (Aldrich, >99%), acetylacetonato-dicarbonyl rhodium(I), ([Rh(acac)(CO)_2_], 99% Strem, Bischeim, France) and chloro(1,5-cyclooctadiene) rhodium(I) dimer ([Rh(COD)Cl]_2_, 98%, Strem, Bischeim, France) were used as received. Styrene (S, 99%, Acros, Saint-Quentin Fallavier, France) was purified by passing through a column of active basic aluminum oxide to remove the stabilizer. 4-[Bis(*p*-methoxyphenyl)phosphino]styrene (BMOPPS) [8] and the RAFT agent 4-cyano-4-thiothiopropylsulfanyl pentanoic acid (CTPPA) [44] were synthesized according to the published procedures. Latexes of core cross-linked nanoparticles functionalized by triphenylphosphine (TPP@CCM) [7] and by bis(*p*-methoxyphenyl)phenylphosphine (BMOPPP@CCM) [8] were prepared as recently described. The characteristics of the used latexes are summarized in Table 1.

### 2.2. Characterization Techniques

NMR: ^1^H NMR and ^31^P NMR spectra were recorded in 5-mm diameter tubes at 297 K in D_2_O using an Avance 400 spectrometer (Bruker Biospin, Wissembourg, France). ^1^H chemical shifts were determined using the residual peak of deuterated solvent as the internal standard and are reported in ppm (δ) relative to tetramethylsilane. ^31^P chemical shifts are reported relative to external 85% H_3_PO_4_. For the CCM and NG characterization, the chemical shift scale was calibrated on the basis of the solvent peak (δ 3.58 for THF-D_8_, 4.79 for D_2_O), and 1,3,5-trioxane was used as an integration reference (δ 5.20).

SEC: Size exclusion chromatography measurements were carried out in THF (with butylhydroxytoluene (BHT) as a flow rate marker) at 20 °C with a flow rate of 1.0 mL·min^−1^. All polymers were analyzed at a concentration of around 5 mg·mL^−1^ after filtration through a 0.45-µm pore size membrane. The separation was carried out on a precolumn and three columns in series (Type Styragel HR1/HR3/HR4). A multi-angle diffusion light scattering (Mini Dawn TriStar Wyatt) was used as detector coupled with a Wyatt Optilab Rex refractometer.

DLS: The intensity average diameters of the latex particles (*D*_z_) and the polydispersity index (PDI) were measured at 25 °C on a Malvern Zetasizer NanoZS. After filtration through a 0.45-µm pore size membrane, deionized water or THF was used to dilute the latex sample. Solutions were analyzed without further filtration to ensure that undesired populations were not removed. Data were analyzed by the general-purpose non-negative least squares (NNLS) method. The typical accuracy for these measurements was 10%–15%.

TEM: The morphological analysis of the copolymer nano-objects was performed with a JEOL JEM 1011 transmission electron microscope equipped with 100 kV voltage acceleration and tungsten filament (Service Commun de Microscopie Electronique TEMSCAN, Centre de Microcaractérisation Raimond Castaing, Toulouse, France). Diluted latex samples were dropped on a formvar/carbon-coated copper grid and dried under vacuum.

### 2.3. Preparation of BMOPPP@NG by One-Pot RAFT Polymerization in Water

This procedure is identical to that previously described for the preparation of TPP@NG [42], except for the use of BMOPPS in place of (4-diphenylphosphino)styrene.

#### 2.3.1. Step 1: Preparation of the HOOCCH_2_CH_2_C(CN)(Me)-(MAA_15_-*co*-PEOMA_15_)-SC(S)SPr Macromolecular RAFT Agent in Water

A stock solution containing ACPA (40 mg·g^−1^) in deionized water containing also NaHCO_3_ (1.5 M) was prepared. A portion of this stock solution (0.1 g, 4 mg ACPA, 0.014 mmol), CTPPA (0.02 g, 0.0722 mmol), MAA (0.096 g, 1.12 mmol), PEOMA (1.04 g, 1.10 mmol) and 4.3 g of deionized water (including the water amount of ACPA solution) were added into a 50-mL flask with a magnetic bar. 1,3,5-Trioxane was also added into the flask as an internal reference for determination of the monomer conversion by ^1^H NMR. The solution in the septum-sealed flask was purged for 45 min with argon and then heated to 80 °C in a thermostated oil bath under stirring. After 120 min, 0.15 mL of solution were taken to determine the monomer conversion and the molar mass of the macroRAFT. The overall monomer molar conversion was about 99%, as determined by ^1^H NMR spectroscopy in DMSO-*d*_6_. The molar mass was analyzed by size exclusion chromatography (SEC) in DMF (experimental *M*_n_ = 15,200 g·mol^−1^; *Ɖ* = 1.11).

#### 2.3.2. Step 2: Preparation of the Nanogels

During Step 1, a biphasic solution of S (0.372 g, 3.6 mmol) and the ACPA stock solution (0.1 g containing 4 mg of ACPA, 0.014 mmol) were purged for 45 min with an argon stream at 0 °C. This solution was quickly injected into the reaction flask, after the completion of Step 1, under argon at 80 °C. After 3 h of stirring, the polymerization was quenched by immersion of the flask into iced water. Then, S (2.5 g, 24 mmol), BMOPPS (0.32 g, 0.91 mmol), DEGDMA (0.13 g, 0.66 mmol) and 100 µL of the ACPA stock solution (4 mg of ACPA, 0.014 mmol) and 5.1 g of deionized water were added. The mixture was purged for 1 h with argon at 0 °C, and the flask was then placed in an oil bath thermostated at 80 °C. After 1 h 30, a 0.5-mL sample was withdrawn for analysis, and the polymerization was quenched by immersion of the flask in iced water. The overall conversion of S and DEGDMA (98%) was determined by ^1^H NMR in THF-*d*_8_ and that of BMOPPS (100%) was measured by ^31^P NMR in THF-*d*_8_. DLS: *D*_h_ = 99 nm (PDI = 0.23) in H_2_O, 236 nm (PDI = 0.23) in THF.

### 2.4. General Procedure for the Phosphine Ligand Complexation to [Rh(acac)(CO)_2_] and [RhCl(COD)]_2_

#### 2.4.1. Loading with [Rh(acac)(CO)_2_]

The [Rh(acac)(CO)(TPP@CCM)] [7], [Rh(acac)(CO)(BMOPPP@CCM)] [8] and [Rh(acac)(CO)-(TPP@NG)] [42] latexes (100% Rh loading to the phosphine functions) were prepared as reported previously. The same procedure was used to prepare the BMOPPP@NG latex 100% loaded with the [Rh(acac)(CO)] fragment, by adjusting the amount of precursor [Rh(acac)(CO)_2_] complex to a slight excess (*ca.* 1.01 equiv) relative to the amount of phosphine functions, as detailed in Table 1. The ^31^P{^1^H} NMR resonances of the different latexes were identical, depending only on the nature of the phosphine function: δ 47.6 (d, *J* = 175 Hz) for TPP and δ 44.5 (d, *J* = 176 Hz) for BMOPPP (162 MHz, CDCl_3_, 298 K).

#### 2.4.2. Loading with [RhCl(COD)]_2_

Latexes metallated with the [RhCl(COD)] fragment were obtained by the same procedure described in Section 2.4.1., by bridge-splitting of the corresponding dimer [RhCl(COD)]_2_. Only the example of TPP@CCM will be detailed. A sample of TPP@CCM latex (0.5 mL, 0.03 mmol of TPP) was diluted with D_2_O (0.5 mL) and swollen by the addition of toluene (0.1 mL). The swelling was very rapid (<1 min upon stirring at room temperature) as confirmed by visual disappearance of the toluene phase. To this sample was added [RhCl(COD)]_2_ (7.7 mg, 0.0155 mmol) in toluene (1 mL), and the resulting mixture was stirred at room temperature for 10 min, during which time the latex color changed to yellow while the supernatant toluene phase became colorless. The aqueous phase was washed with toluene (2 × 1 mL) under argon to remove any excess of the Rh complex; both toluene washings were colorless. The resulting [RhCl(COD)(TPP@CCM)] latex was collected after decantation for further NMR studies. ^31^P{^1^H} NMR (162 MHz, CDCl_3_, 298K): δ 29.4 (d, *J* = 147 Hz). The same procedure was also used to load the other latexes with [RhCl(COD)]. ^31^P{^1^H} NMR (162 MHz, D_2_O, 298K) for [RhCl(COD)(BMOPPP@CCM)]: δ 26.9 (d, *J* = 149 Hz). As for the case of the 100% [Rh(acac)(CO)]-loaded latexes, the ^31^P NMR spectrum of all 100% [RhCl(COD)]-loaded latexes were independent of the type of latex (CCM or NG), depending only on the type of phosphine function bonded to rhodium. Additional CCM and NG latexes loaded with a different mole % of the Rh precursor for exchange studies, as detailed in the Results and Discussion section, were prepared by the same procedure adjusting the amount of rhodium complex to the desired fraction.

### 2.5. Interparticle Metal Exchange Study Involving 100% [Rh(acac)(CO)]-Loaded and Rh-Free TPP@NG

These experiments were carried out as previously described for the TPP@CCM particles [43].

#### 2.5.1. At the Natural pH

The two starting latexes were prepared independently by diluting 0.5 mL of TPP@NG (0.034 mmol of TPP, 0.033 mmol of MAA) with D_2_O (0.43 mL). One of these two samples was charged with [Rh(acac)(CO)_2_] (100% loading), as described above. The second sample was swollen with the same amount of toluene used for the first sample in order to obtain the same concentration of particles. Equivalent volumes of these two samples were then directly mixed in an NMR tube under argon, and the resulting mixture was monitored by ^31^P{^1^H} NMR spectroscopy (see the Results and Discussion).

#### 2.5.2. Under Basic Conditions

This procedure is identical to that described in Section 2.5.1., except that the two starting latexes, one of which was charged with [Rh(acac)(CO)_2_] (100% loading), were prepared from 0.5 mL of TPP@NG (0.034 mmol of TPP, 0.034 mmol of MAA) with D_2_O (0.43 mL). Forty microliters of a concentrated NaOH solution (10 N, 0.4 mmol) were added to each starting latex to adjust the final pH to 13.6, before mixing in the NMR tube.

### 2.6. Interparticle Double Exchange Study Involving 100% [Rh(acac)(CO)]-Loaded BMOPPP-Functionalized Polymer Latex and 100% [RhCl(COD)]-Loaded TPP-Functionalized Polymer Latex

#### 2.6.1. Using the CCM Particles

The TPP@CCM and BMOPPP@CCM latexes (0.5 mL, 0.03 mmol of FS) were independently diluted into D_2_O (0.5 mL). The TPP@CCM sample was loaded with [Rh(COD)Cl]_2_, and the BMOPPP@CCM sample was loaded with [Rh(acac)(CO)_2_], as described above. Equivalent volumes of the two latexes were then directly mixed in an NMR tube under argon. The resulting solution was monitored by ^31^P{^1^H} NMR spectroscopy (see the Results and Discussion).

#### 2.6.2. Using the NG particles.

This procedure is identical to that described above for the CCM particles, using [Rh(acac)(CO)(BMOPPP@NG)] and [RhCl(COD)(TPP@NG)] samples.

## 3. Results and Discussion

### 3.1. Synthesis and Characterization of BMOPP@NG

**Figure 3:**
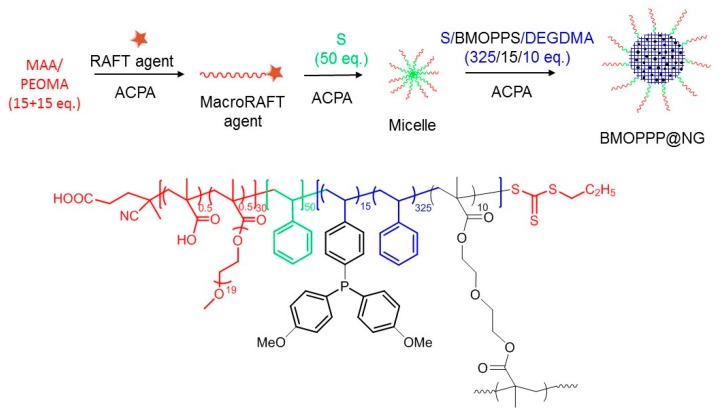
Synthetic scheme and structure of BMOPPP@NG. MAA, methacrylic acid; PEOMA, poly(ethylene oxide) methyl ether methacrylate; ACPA, 4,4′-azobis(4-cyanopentanoic acid); DEGDMA, di(ethylene glycol) dimethacrylate.

The new BMOPP@NG polymer was synthesized in the same way as the previously-reported TPP@NG [42] (see Figure 3) and is quite similar in structure, size and composition to that polymer (see the details in Table 1). The only difference is the use of 4-[bis(*p*-methoxyphenyl)phosphino]styrene (BMOPPS) in place of 4-(diphenylphosphino)styrene (DPPS) as the functional monomer during the second step of the synthesis (details in Section 3). It is obtained directly as a stable latex with *ca.* 25% weight of polymer. The self-dissociation of the shell methacrylic acid functions establishes the latex pH as *ca.* 4–5, depending on the subsequent dilution. We shall refer to this as the “natural pH”. All polymers used in the present study, including the previously-reported TPP@CCM and BMOPPP@CCM, have the same composition in terms of the number of hydrophilic monomers per RAFT agent in the outer shell (15 methacrylic acid (MAA) and 15 poly(ethylene oxide) methyl ether methacrylate) and functional monomer (15), di(ethylene glycol) dimethacrylate (DEGDMA) cross-linker (10) and styrene (375) in the hydrophobic core. While the synthesis of TPP@CCM and TPP@NG could also be accomplished with a greater amount of functional monomer [7,9,42], the BMOPPP-functionalized particle latex was limited to 15 equivalents of functional monomer per RAFT agent because of the limited solubility of BMOPPS in styrene.

**Table 1:** Polymer latexes used in this study ^a^. PDI, polydispersity index; DPPS, 4-(diphenylphosphino)styrene.

Polymer	FS ^b^	*D*_z_ (nm)/PDI		Solid (%)	[FS] (mmol/mL)	Reference
		H_2_O	THF			
TPP@CCM	DPPS	100/0.28	163/0.07	25.6	0.060	[7]
BMOPPP@CCM	BMOPPS	81/0.16	216/0.20	25	0.058	[8]
TPP@NG	DPPS	86/0.20	188/0.15	27.8	0.068	[42]
BMOPPP@NG	BMOPPS	99/0.23	236/0.23	27.9	0.058	This work

^a^ CCM: HOOCCH_2_CH_2_C(CN)(Me)-[MAA_15_-*co*-PEOMA_15_]-*b*-[S_285_-*co*-(FS)_15_]-*b*-[S_90_-*co*-DEGDMA_10_]-SC(S)SPr; NG: HOOC-CH_2_CH_2_C(CN)(Me)-[MAA_15_-*co*-PEOMA_15_]-*b*-S_50_-*b*-[S_325_-*co-*(FS)_15_-*co*-DEGDMA_10_]; ^b^ FS = functionalized styrene.

**Figure 4:**
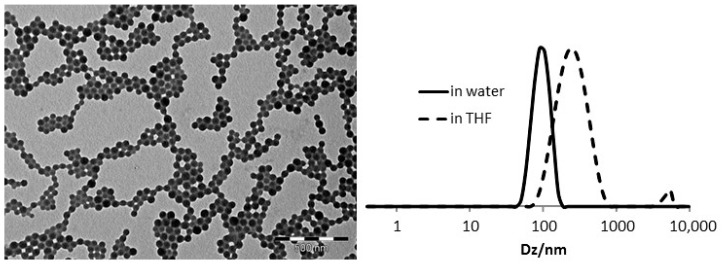
TEM image (**left**) and DLS response in water and THF solutions (**right**) for BMOPP@NG.

The TEM and DLS characterization of the new BMOPPP@NG polymer confirms the good control throughout the synthesis, with the nanoreactors having a spherical shape, small and regular dimensions and narrow size distributions; see Figure 4. The particle diameter increases going from water to THF solution because THF is a good solvent for the polystyrene-based core. These properties are very similar to those of the TPP@NG and CCM analogues; see Table 1.

The NMR properties are also similar to those previously established for the corresponding TPP@NG, TPP@CCM and BMOPPP@CCM analogues. Because of the large dimensions and water insolubility of the particle core, the ^1^H NMR spectrum of BMOPPP@NG in D_2_O reveals only the resonances of the PEO chains (Figure S1), while the ^31^P NMR spectrum is silent. The outer shell backbone protons (MAA CH_3_, CH_2_ and CH protons and PEOMA CH_2_ and CH protons) are also invisible, because the shell backbone is not solvated and remains solidary with the hydrophobic core. All protons become visible, however, in THF-D_8_, showing that the low cross-linking density (one cross-linker for 40 monomers in the hydrophobic core) confers sufficient mobility to the full macromolecule, like for the corresponding more flexible CCM. The core phosphine functions become visible in the ^31^P{^1^H} NMR spectrum with a single resonance at δ −11.5. This is the same chemical shift observed for the resonance of BMOPPP@CCM [8] and is slightly upfield relative to the resonances of TPP@CCM [7] and TPP@NG [42] (δ −8.3). The addition of toluene, compatible with the particle polystyrene core, to the latex results in nanoparticle swelling, the resonances of the core H and P nuclei becoming observable in the NMR spectra (Figure S1). The ^31^P{^1^H} resonance is observed at δ −9.7 under these conditions. Similar to the behavior previously reported for the CCM and TPP@NG particles [7,8,42], the outer shell PEO resonances are split into two sets upon toluene swelling: a sharper one associated with the more mobile water-solvated PEO chains and a broader one associated with less mobile toluene-solvated chains inside the hydrophobic core. This double population results from the peculiar structuring of the core-shell interface, as previously discussed in detail [43]. Line deconvolution of the stronger PEO methylene resonance (Figure S2) yields ratios for the water-solvated and toluene-solvated PEO chains of 22.0:78.0, showing that toluene swelling makes the major fraction of the PEO chains compatible with the hydrophobic core. Similar results were obtained for the other related particles (30.3:69.7 distribution for TPP@CCM [7], 23.2:76.8 for BMOPPP@CCM [8] and 37.9:62.1 for TPP@NG [42]). Rough integration of the free toluene resonances (accuracy is limited because of the overlap with the broader polymer resonances; see Figure S1) allows estimating the incorporation of 760–860 molecules of toluene per chain for the swollen BMOPPP@NG (*cf.* 770–950 for TPP@CCM [7], 730–810 for BMOPPP@CCM [8] and 450–520 for TPP@NG [42]). The two estimates in each case correspond to the integration of the aromatic and methyl proton resonances. The relatively low cross-linking density does not introduce dramatic constraints to significantly alter the polymer swelling capacity and the chain mobility.

### 3.2. Metal Coordination inside the Nanoreactors

**Figure 5:**
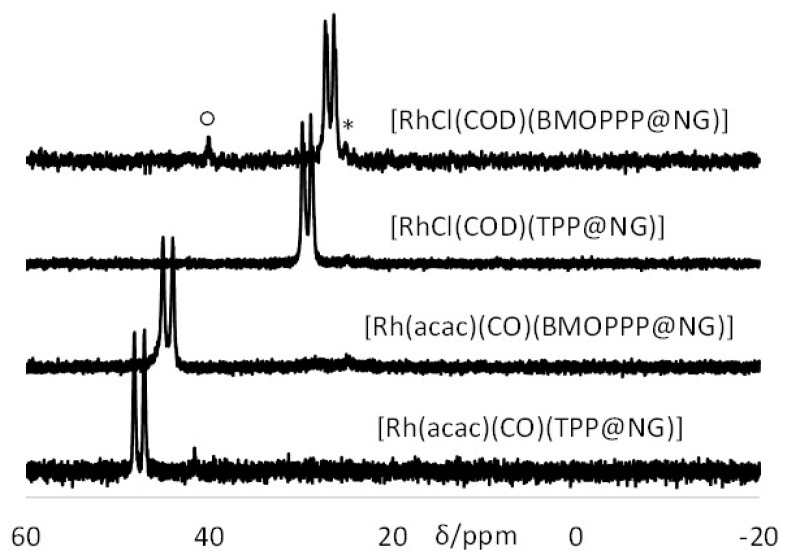
^31^P{^1^H} NMR spectra of TPP@NG and BMOPP@NG 100% loaded with [Rh(acac)(CO)_2_] or [RhCl(COD)]_2_ in water after swelling with toluene. The starred resonance corresponds to a small amount of oxidized phosphine. The resonance marked with a circle belongs to an unknown impurity.

Loading the toluene-swollen BMOPPP@NG with [Rh(acac)(CO)_2_] (1 equiv per P atom) results in CO replacement and coordination of the Rh centers to all polymer phosphine functions according to Equation (2) (see also Figure 2A), replacing the ^31^P NMR signal of the free phosphine at δ −9.7 with a doublet resonance at δ 44.5 (d, *J* = 172 Hz) for the Rh-bonded phosphine in [Rh(acac)(CO)-(BMOPPP@NG)]; see Figure 5. This resonance corresponds closely in the chemical shift and Rh coupling to that of the related molecular compound [Rh(acac)(CO){P(C_6_H_4_-4-OMe)_3_}] (δ 43.5, *J*_PRh_ = 175.6 Hz) [45]. It is also essentially identical to that previously reported for [Rh(acac)(CO)(BMOPPP@CCM)] [8] (recalled in the SI, Figure S3). By analogy with the behavior previously described for the CCM [7,8], loading with only ½ equivalent of metal complex, therefore leaving 50% of non-coordinated phosphine functions, yields silent spectra, because the rate of the self-exchange process (Equation (3)) results in resonance coalescence at room temperature. The addition of [Rh(acac)(CO)_2_] to TPP@NG, as previously described [42], yields a resonance δ 47.6 (d, *J* = 175 Hz) for [Rh(acac)(CO)(TPP@NG)]. Since this resonance is a useful reference for the new coordination chemistry studies described in the present contribution, it is also displayed in Figure 5, and the related resonance of [Rh(acac)(CO)-(TPP@CCM)] [7] is shown in Figure S3. The chemical shift and Rh coupling parameters for all resonances are also collected for convenience in Table 2.

[Rh(acac)(CO)_2_] + BMOPPP@NG → [Rh(acac)(CO)(BMOPPP@NG)] + CO (2)

[Rh(acac)(CO)(BMOPPP@NG)] + BMOPPP@NG ⇄ BMOPPP@NG + [Rh(acac)(CO)(BMOPPP@NG)]
(3)

**Table 2:** ^31^P{^1^H} NMR properties of the 100% Rh-loaded polymers.

Polymer	M = Rh(acac)(CO)		M = RhCl(COD)	
	δ/ppm (*J*/Hz)	Reference	δ/ppm (*J*/Hz)	Reference
[M(TPP@CCM)]	47.5 (175)	[7]	29.3 (149)	This work
[M(BMOPPP@CCM)]	44.5 (176)	[8]	26.8 (151)	This work
[M(TPP@NG)]	47.6 (175)	[42]	29.3 (150)	This work
[M(BMOPPP@NG)]	44.5 (172)	This work	26.8 (150)	This work

In addition to loading with the [Rh(acac)(CO)] fragment, we now report that all nanoreactors can also be loaded with the [RhCl(COD)] fragment (COD = η^4^-1,5-cyclooctadiene). This was accomplished by adding the corresponding dichloro-bridged dimer after swelling with toluene (Equation (4); see also Figure 2B). The coordination process is rapid and quantitative, illustrating that mass transport of the metal precursor to the particle core is a facile process as in the case of [Rh(acac)(CO)_2_]. The ^31^P{^1^H} NMR spectra of the resulting 100% loaded NG polymers are shown in Figure 5, and those of the corresponding CCM polymers, which are essentially identical, are shown in the SI (Figure S3). The resonances of [RhCl(COD)(TPP@CCM)] and [RhCl(COD)(TPP@NG)], at δ 29.3 (*J*_PRh_ = 149 Hz) for both samples, are close in position to those reported for the molecular [RhCl(COD)(PPh_3_)] complex (δ 31.5, *J*_PRh_ = 152 Hz) and for other polymer-supported samples (δ 30.9, *J*_PRh_ = 147 Hz) [46]. The resonances of [RhCl(COD)(BMOPPP@CCM)] and [RhCl(COD)-(BMOPPP@NG)], at δ 26.8 (*J*_PRh_ = 151 Hz) for both samples are close to those of the closely-related molecular [RhCl(COD){P(*p*-C_6_H_4_OMe)_3_] complex (δ 27.0, *J*_PRh_ = 149.4 Hz) [47].

½ [RhCl(COD)]_2_ + L@Pol → [RhCl(COD)(L@Pol)] (L = TPP or BMOPPP; Pol = CCM or NG) (4)

**Figure 6:**
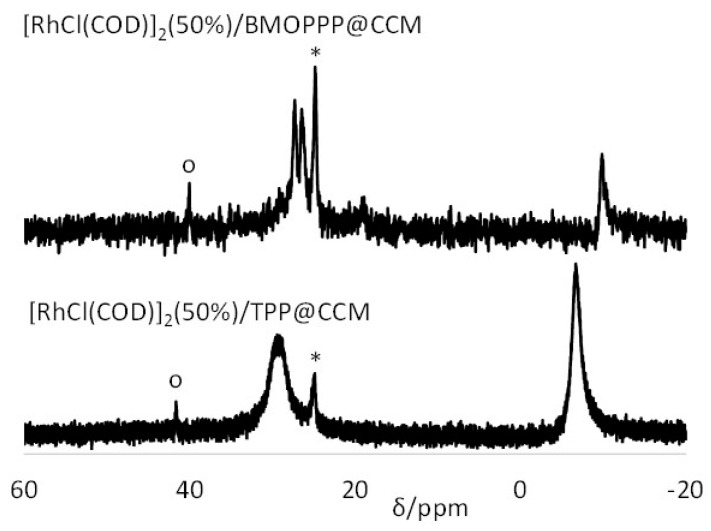
^31^P{^1^H} NMR spectra of TPP@CCM and BMOPPP@CCM 50% loaded with [RhCl(COD)]_2_ in water after swelling with toluene. The corresponding TPP@NG and BMOPPP@NG polymers gave the same result. The starred resonances correspond to the oxidized phosphine impurity. The resonance marked with a circle is an impurity of unknown nature.

Loading the polymers with only 50% of the [RhCl(COD)]_2_ amount required to saturate all phosphine ligands led to the spectra shown in Figure 6. In these cases, contrary to the 50% [Rh(acac)(CO)]-loaded samples, the resonances of both the free and the coordinated ligands are clearly visible, indicating that the self-exchange process (Equation (5)) is slower. The resonances are a bit broader in the case of the [RhCl(COD)]_2_/TTP combination, to the point that the P–Rh coupling for the coordinated phosphine resonance is no longer discernible, indicating that the self-exchange is slightly faster for the polymer-anchored TPP than for BMOPPP under the same conditions.

[RhCl(COD)(L@Pol)] + L@Pol ⇄ L@Pol + [RhCl(COD)(L@Pol)] (L = TPP or BMOPPP; Pol = CCM or NG)
(5)

As previously shown [43], the [Rh(acac)(CO)]-loaded latexes are unaffected by raising the pH: the ^31^P{^1^H} NMR resonance at pH 13.6 is identical to that recorded at the natural pH and did not change with time. On the other hand, the [RhCl(COD)]-loaded latexes showed instability at high pH, the possible reason being a reaction between the Rh–Cl bond and OH^−^. In our previous study, we have shown that ions, such as Cl^−^ and OH^−^, accompanied by the Na^+^ counterion, are vectorized very rapidly from the aqueous solution to the hydrophobic polymer core [43].

### 3.3. Interparticle Metal Migration for [Rh(acac)(CO)]-Loaded TPP@NG

Metal migration between different TPP@CCM nanoreactors, probed by ^31^P NMR monitoring of an equimolar mixture of TPP@CCM and [Rh(acac)(CO)(TPP@CCM)], *i.e.*, 0% and 100% [Rh(acac(CO)]-loaded latexes, was recently shown to be very fast (<5 min) at the natural pH and to be dramatically retarded (*ca.* 10 h) at pH 13.6 [43]. The same migration has now been investigated using the NG polymer architecture. Mixing equimolar amounts of TPP@NG and [Rh(acac)(CO)-(TPP@NG)] at the natural pH resulted in the immediate observation of a silent ^31^P NMR spectrum, no matter how rapidly the spectrum was recorded after mixing. This result is identical to that obtained using the TPP@CCM latex showing, though only qualitatively, that placing the phosphine functions inside the cross-linked area (NG particles), relative to the flexible arms outside the cross-linked core (CCM particles; see Figure 1), has no dramatic effect on the ligand exchange process. Quite clearly, the very low cross-linking density in these NG particle cores does not block the associative exchange on the Rh centers of the phosphine ligand bonded to one particle core with a free phosphine ligand bonded to another particle core.

**Figure 7:**
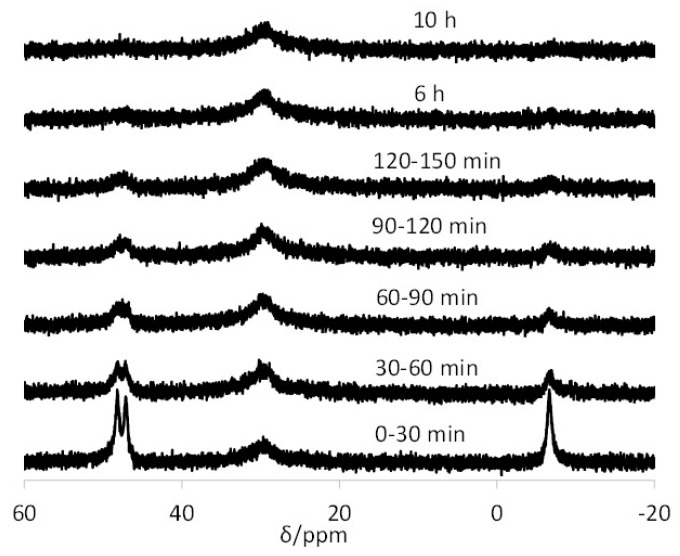
^31^P{^1^H} NMR spectra of the latex obtained by mixing equimolar amounts of TPP@NG and [Rh(acac)(CO)(TPP@NG)] at pH 13.6. The data for each spectrum were collected for 30 min; the time intervals for data collection from the time of mixing are indicated on each spectrum.

Upon repeating the experiment at pH 13.6, the migration dramatically slows down; see Figure 7. This behavior is also identical to that recorded for the related TPP@CCM, the timescale for complete exchange (*ca.* 10 h) being approximately the same [43]. The new resonance appearing at δ *ca.* 29.5, also observed in the TPP@CCM experiment, belongs to the bis-phosphine derivative [Rh(OH)(CO)(TPP@NG)_2_], produced as shown in Equation (6). This was demonstrated in our previous contribution by a combined NMR, IR and DLS study [43]. An analogous reaction where NaCl was used in place of NaOH led to the quantitative generation of [RhCl(CO)(TPP@CCM)_2_] according to the same stoichiometry. In that study, it was also unambiguously proven that the core-core contact is totally blocked at pH 13.6, and thus, the residual metal migration under basic conditions cannot result from a direct associative phosphine exchange. It is most probably related to the migration of the Rh ion as a molecular complex from particle to particle via the continuous aqueous phase. The new results obtained with the TPP@NG particles are consistent with this view, because the metal migration mechanism through the continuous aqueous phase should not be significantly affected by the polymer core architecture.

[Rh(acac)(CO)(TPP@Pol)] + TPP@Pol + NaX → [RhX(CO)(TPP@Pol)_2_] + Na(acac) (X = OH, Cl; Pol = CCM or NG) (6)

### 3.4. Interparticle Cross-Migration

**Figure 8:**
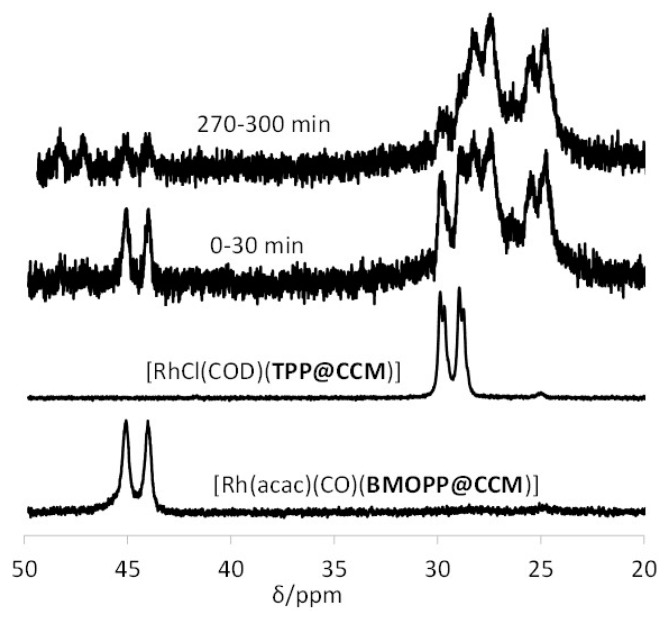
^31^P{^1^H} NMR spectra of the latex obtained by mixing equimolar amounts of [Rh(acac)(CO)(BMOPPP@CCM)] and [RhCl(COD)(TPP@CCM)]. The data for each spectrum were collected for 30 min; the time intervals for data collection from the time of mixing are indicated on each spectrum. The spectra of the starting latexes are also included for comparison.

In order to eliminate the problem of ^31^P NMR resonance coalescence by the intraparticle phosphine exchange (e.g., as in Equations (1) and (3)), which affects all polymers loaded with the substitutionally more labile [Rh(acac)(CO)] fragment, a cross-migration experiment with fully-metal-loaded polymers has been conceived of, as shown in Equation (7). In addition, since the phosphine exchange on square planar Rh^I^ complexes is known to follow an associative mechanism with a rate law that is first order in the Rh complex and first order in the entering ligand, reducing the free phosphine concentration to zero in the fully-loaded polymers should slow down the exchange and facilitate monitoring of the reaction progress. In principle, the spectroscopic monitoring should show evolution from the two starting materials on the left-hand side of the equation toward all four possible metal-ligand combinations with an approximately statistical (25:25:25:25) distribution. An analogous study of the molecular version of this reaction (using [Rh(acac)(CO)[P(*p-*C_6_H_4_OMe)_3_}] and [RhCl(COD)(PPh_3_)] in CDCl_3_) has recently been reported [47]. The experiment carried out using the CCM systems gave the salient results shown in Figure 8.

[Rh(acac)(CO)(BMOPPP@Pol)] + [RhCl(COD)(TPP@Pol)] ⇄ [RhCl(COD)(BMOPPP@Pol)] + [Rh(acac)(CO)(TPP@Pol)] (Pol = CCM or NG)
(7)

**Scheme 1:**
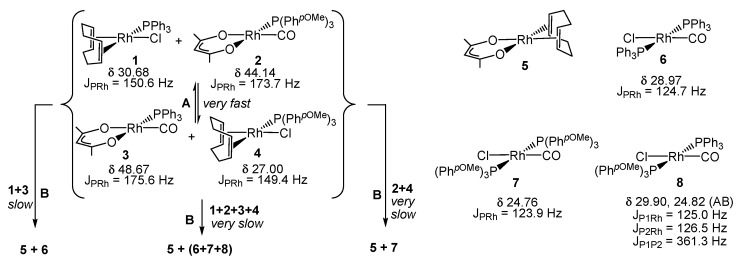
Ligand exchange processes occurring between [Rh(acac)(CO)[P(*p-*C_6_H_4_OMe)_3_}] and [RhCl(COD)(PPh_3_)] in CDCl_3_ [47].

In the spectrum recorded within the first 30 min, the two doublets of the starting polymers at δ 44.5 and 29.3 can be clearly observed, while those of the expected products (*cf.* Figure 5) are not. However, there are unexpectedly two additional major doublet resonances at δ 27.8 (*J*_PRh_ = *ca.* 115 Hz) and 25.1 (*J*_PRh_ = *ca.* 115 Hz). With time, the starting compound resonances decrease and the resonance of one of the expected products, [Rh(acac)(CO)(TPP@CCM)], is observed at δ 47.5 (*cf.* Table 2), converging toward the expected equivalent intensity relative to the starting compound. The resonance of the other final product, [RhCl(COD)(BMOPPP@CCM)], expected at δ 26.8, is not observed because it is overshadowed by the other two major resonances. The resonance intensity evolution indicates rapid equilibration (*t*_1/2_ *ca.* 1 h) of the metal complexes between the two types of polymers to the expected statistical 1:1 mixture, whereas the two major doublet resonances at δ 27.8 and 25.1 are produced immediately and do not significantly evolve with time. The assignment and the genesis of these two unexpected resonances is apparent on the basis of the results of the previously investigated homogeneous system, which are recalled in Scheme 1 [47].

**Scheme 2:**
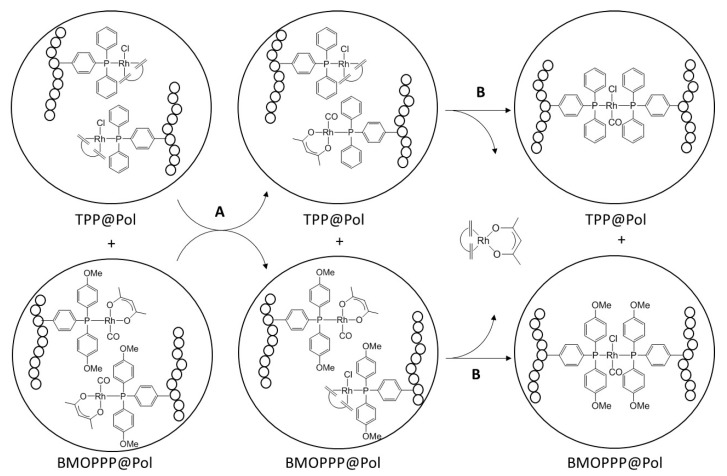
Ligand exchange processes occurring between [RhCl(COD)(TPP@Pol)] and [Rh(acac)(CO)(BMOPPP@Pol)] (Pol = CCM or NG). The study was carried out by mixing the polymer latexes (*i.e.*, water solutions), after swelling with toluene and charging with the metal complexes (see Materials and Methods).

The phosphine scrambling process leading from a 50:50 mixture of **1** and **2** to a statistical 25:25:25:25 distribution of Compounds **1**, **2**, **3** and **4** (Process A) is very fast, equilibrium being reached within the time of recording the first spectrum (<5 min). A much slower process (Process B, >24 h) then follows, leading to scrambling of the other ligands with quantitative conversion into Compounds **5**, **6**, **7** and **8**. Simplified ligand scrambling processes in Step B were observed upon mixing **1** and **3**, quantitatively leading to **5** and **6**, or mixing **2** and **4**, leading to **5** and **7**. Comparison of the chemical shifts and coupling constants with the products in Scheme 1 allows rationalization of the spectral evolution in Figure 8, as shown in Scheme 2, therefore assigning the two doublet resonances at δ 27.8 and 25.1 to [RhCl(CO)(TPP@CCM)_2_] and [RhCl(CO)(BMOPPP@CCM)_2_]. The same compound [RhCl(CO)(TPP@CCM)_2_] has been previously obtained according to Equation (6) (broad resonance at *ca*. δ 28.5) [43].

One of the most important differences between the homogeneous and polymer-confined reactions is that in the latter case, each type of phosphine ligand is restrained within its own polymer core. Therefore, the polymer-grafted equivalent of the mixed phosphine complex (**8**) is inaccessible, except through core interpenetration. Since a complex feature attributable to a polymer-confined mixed phosphine species equivalent to **8** is not observed in Figure 8, particle interpenetration does not occur to a great extent. Indeed, our recent DLS study of the 50% [Rh(acac)(CO)]-loaded TPP@CCM in the presence of NaOH or NaCl (Equation (6)) revealed that only 1–2 events per particle core (containing *ca.* 10^5^ Rh atoms) lead to particle coupling, while all other events involve two phosphine ligands located within the same particle [43]. Thus, the ligand exchange (Equation (7)) between complexes located within the same polymer core occurs much more frequently than between complexes located in different cores, leading to the homoleptic bis(phosphine) complexes as the only observable products.

On the basis of our recent study of metal migration in the CCM polymers [43], confirmed by analogous findings in the NG polymers (*vide supra*), Process A probably occurs by direct phosphine exchange during transient interpenetration with core-core contact, the exchange being either associative because of the presence of a minor amount of residual non-metallated phosphine ligands (incomplete polymer loading by the metal complex) or dissociative. It is also possible to envisage an associative mechanism via chloro-bridged dimetallic intermediates, as discussed in our previous investigation of the homogeneous version of the same reaction [47]. A cross-migration mechanism involving migration of molecular species through the continuous aqueous phase is ruled out because it would occur on a timescale of several hours (*cf.* Figure 7). Unfortunately, it was not possible to verify the exchange rate reduction at higher pH because of the instability of the [RhCl(COD)(L@CCM)] functions (L = TPP or BMOPPP) in a basic medium (*vide supra*). The comparison between the timescale of the cross-exchange for the molecular complexes in homogeneous solution (<5 min) and in the nanoreactor core (*ca.* 1 h) reflects the additional requirement for transient particle interpenetration with core-core contact.

Unlike Step A, which is slower for the reaction involving the nanoreactor confined complexes, Step B is faster for the reaction in the polymer scaffold (t_1/2_ < 30 min) relative to the homogeneous solution (>24 h), as shown by the immediate prominence of the resonances of [RhCl(CO)(TPP@CCM)_2_] and [RhCl(CO)(BMOPPP@CCM)_2_] in the NMR spectrum (Figure 8). This difference must result from the higher local concentration of the rhodium complexes, after their generation from Step A, in the confined polymer core environment. Closer inspection of the first spectrum recorded in the 0–30 min interval, in fact, shows the prominence of the final products in the absence of a significant amount of the intermediate resulting from Step A, demonstrating that Step B is much faster than Step A, whereas the opposite is true for the molecular version in homogeneous solution.

A final interesting observation is the presence and persistence of the intermediate monophosphine complexes resulting from Step A at equilibrium. The stoichiometry should in principle lead to total disappearance of these complexes, since Step B is quantitative in the molecular version. The rationalization of this result is based on the polymeric structure: formation of the bis-phosphine complexes creates additional chain cross-links (see Scheme 2), rigidifying the polymer core and inevitably leaving a few [Rh(acac)(CO)(L@CCM)] and [RhCl(COD)(L@CCM)] functions (L = TPP, BMOPPP) physically incapable of finding a suitable partner with which to react.

**Figure 9:**
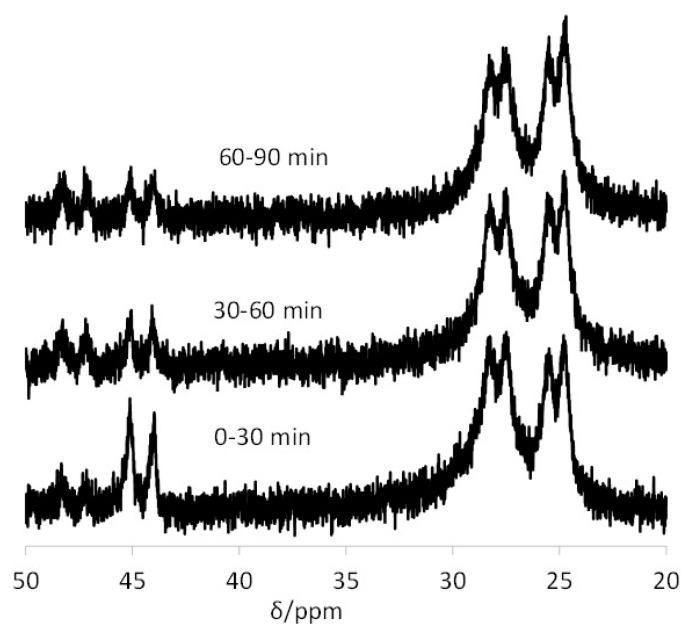
^31^P{^1^H} NMR spectra of the latex obtained by mixing equimolar amounts of [Rh(acac)(CO)(BMOPPP@NG)] and [RhCl(COD)(TPP@NG)]. The data for each spectrum were collected for 30 min; the time intervals for data collection from the time of mixing are indicated on each spectrum.

Monitoring the same double exchange process in the NG polymers, the relevant features of which are summarized in Figure 9, shows the same salient features: rapid development of prominent resonances for the two homoleptic bis(phosphine) complexes and persistence of the two [Rh(acac)(CO)]-supported complexes in small amounts and in an approximately 1:1 ratio. In this case, the resonance of the [RhCl(COD)(TPP@NG)] starting material was not clearly visible in the first spectrum, which may be caused by an incomplete loading of the TPP@NG polymer with the [RhCl(COD)]_2_ complex, yielding a broader resonance for these functions (*cf.* Figure 6).

## 4. Conclusions

The present study has provided additional information on the interparticle metal migration process involving latexes of the amphiphilic core-shell polymers shown in Figure 1. For the NG particles, where the phosphine ligands are buried inside the cross-linked hydrophobic core, rapid metal migration still occurs through direct associative phosphine exchange during particle interpenetration with core-core contact. Quite evidently, the low cross-linking density (one cross-linking monomer for 40 hydrophobic monomers) still permits sufficient flexibility to allow at least a few of the Rh complexes in one particle core to come into direct contact with at least a few free phosphine functions of another polymer core. The coordinated and free phosphine functions that are located in the innermost part of the cores can then equilibrate via intraparticle exchange processes. The rate of the metal migration, however, is much slower at high pH where the shell carboxylic functions are deprotonated and very close to that previously observed for the CCM particles. A cross-migration study was made possible by using nanoreactors containing two different phosphines, TPP and BMOPPP, fully loaded with two different metal fragments, [Rh(acac)(CO)] and [RhCl(COD)], thus avoiding the presence of free phosphine functions, which lead to signal coalescence by rapid intraparticle metal exchange. This study was carried out both with the CCM and the NG polymer architectures, which required the synthesis of the previously unreported BMOPP@NG polymer. The study has revealed a relatively rapid metal migration by phosphine exchange (*ca.* 30 min), though this exchange is much slower than the equivalent homogeneous process for the molecular complexes. On the other hand, a rapid scrambling of the other ligands, mostly within the same nanoreactor core, occurs very rapidly after the metal migration leading to the polymer-linked complexes [RhCl(CO)(L@Pol)_2_] (Scheme 2). This step occurs much faster than the corresponding homogeneous process for the molecular compounds. We have therefore demonstrated that a reaction between species that are confined within different nanoreactor cores occurs more slowly than the equivalent reaction of molecular species under homogeneous conditions. This is expected, because it requires nanoreactor collision and core-core contact, in addition to collision of the reactants while the nanoreactors are interpenetrated. On the other hand, a reaction between species that are confined within the same nanoreactor core occurs more rapidly than the equivalent reaction of molecular species under homogeneous conditions.

## Supplementary Materials

Supplementary materials can be found at www.mdpi.com/2073-4360/8/2/26/s1.

## Abbreviations

The following abbreviations are used in this manuscript:

acac: acetylacetonato ligand
BMOPPP: bis(*p*-methoxyphenyl)phenylphosphine
CCM: core-cross-linked micelle
COD: η^4^-1,5-cyclooctadiene ligand
NG: nanogel
TPP: triphenylphosphine
