# Supplementary material for: Coordination Chemistry inside Polymeric Nanoreactors: Metal Migration and Cross-Exchange in Amphiphilic Core-Shell Polymer Latexes
Source: Polymers (Basel). 2016 Jan 22;8(2):26. doi: 10.3390/polym8020026 (PMC6432602; doi:10.3390/polym8020026)
Supplement: Supplementary file 1 [file polymers-08-00026-s001.pdf]

Supplementary Maretials: Coordination Chemistry Inside Polymeric Nanoreactors: Metal Migration and Cross-Exchange in Amphiphilic Core-Shell Polymer Latexes

Si Chen, Eric Manoury, Florence Gayet and Rinaldo Poli

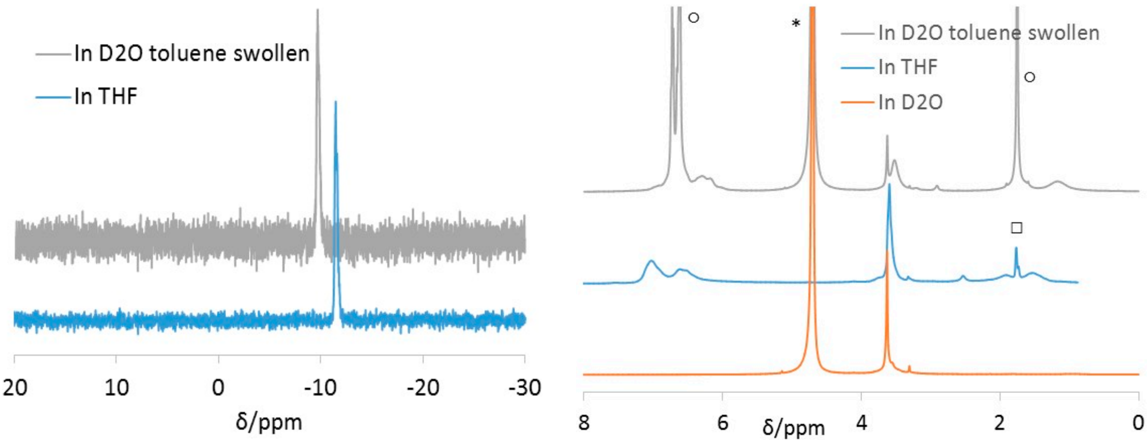

Figure S1. ^{1}H-NMR (**left**) and ^{31}P-NMR (**right**) spectra of the **BMOPPP@NG** latex. The spectra in D_2O were recorded directly after addition of D_2O for the instrument lock to the latex (either toluene-swollen or not). The starred resonance at δ 4.7 belongs to water. Those at δ 3.63 (strong) and 3.30 (weak) belong to the CH_2 and CH_3 protons, respectively, of the PEOMA–$(OCH_2CH_2)_{19}$-OCH_3 protons. The spectra in THF-D_8 were taken after drying the latex to a solid residue and dissolution in the NMR solvent. The resonance marked with a square at δ 1.76 belongs to the solvent, with the second one at ca. δ 3.6 being masked by the PEOMA proton resonance. The resonances marked with a circle belong to the toluene molecules hosted in the swollen polymer core.

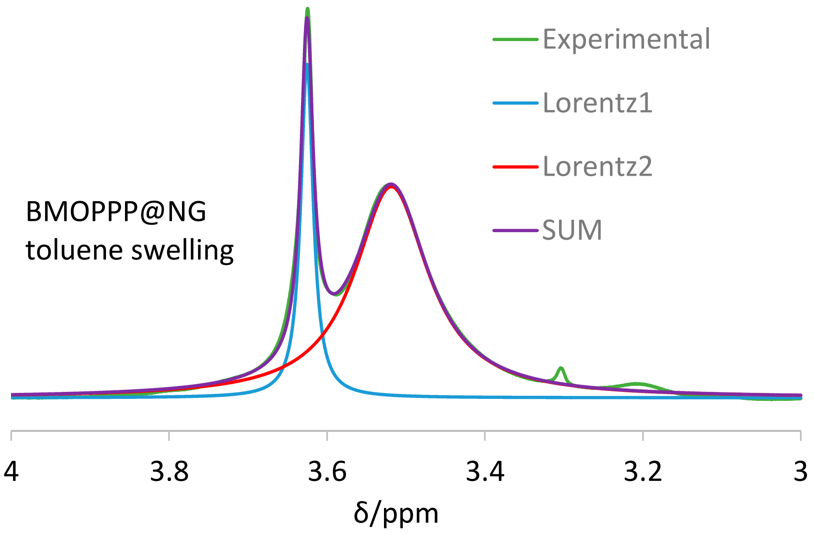

Figure S2. Excerpt of the ^{1}H-NMR spectra of **BMOPP@NG** after swelling with toluene in the region of the methylene and methoxy PEOMA proton resonances, and deconvolution of the CH_2 resonance.

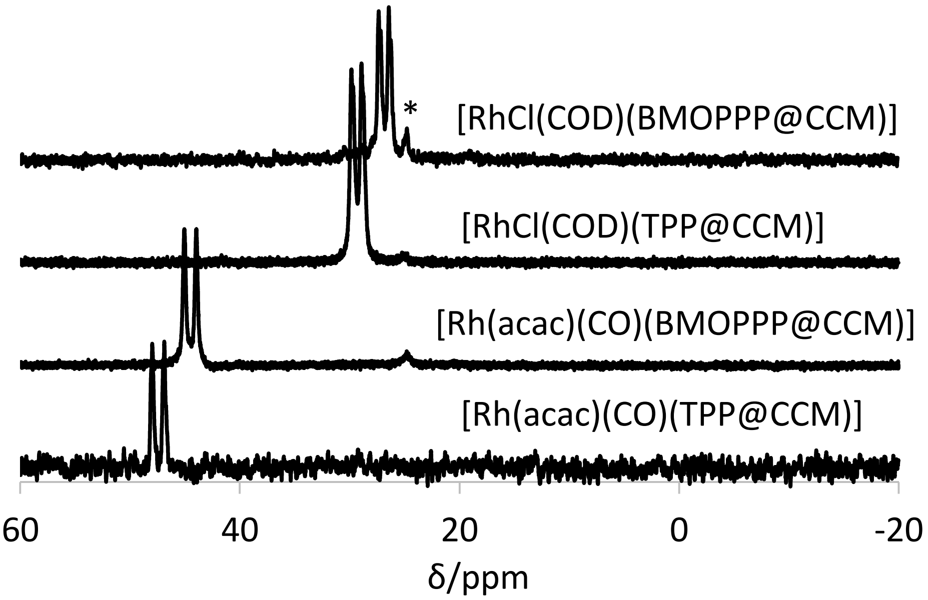

Figure S3. $^{31}P\{^1H\}$ NMR spectra of the **TPP@CCM** and **BMOPPP@CCM** latexes in D_2O after swelling the polymer core with toluene and 100% loading with [Rh(acac)$(CO)_2$] or [RhCl(COD)$]_2$. The starred resonance corresponds to a small amount of oxidized phosphine.

© 2016 by the authors; licensee MDPI, Basel, Switzerland. This article is an open access article distributed under the terms and conditions of the Creative Commons by Attribution (CC-BY) license (http://creativecommons.org/licenses/by/4.0/).
